# Graphene–Graphite Polyurethane Composite Based High‐Energy Density Flexible Supercapacitors

**DOI:** 10.1002/advs.201802251

**Published:** 2019-02-13

**Authors:** Libu Manjakkal, William Taube Navaraj, Carlos García Núñez, Ravinder Dahiya

**Affiliations:** ^1^ Bendable Electronics and Sensing Technologies (BEST) Group School of Engineering University of Glasgow G12 8QQ Glasgow UK; ^2^ SUPA Institute of Thin Films Sensors and Imaging School of Computing Engineering and Physical Sciences University of the West of Scotland PA12BE Paisley Scotland UK

**Keywords:** energy autonomy, flexible supercapacitors, graphite, photovoltaic cells, wearable systems

## Abstract

Energy autonomy is critical for wearable and portable systems and to this end storage devices with high‐energy density are needed. This work presents high‐energy density flexible supercapacitors (SCs), showing three times the energy density than similar type of SCs reported in the literature. The graphene–graphite polyurethane (GPU) composite based SCs have maximum energy and power densities of 10.22 µWh cm^−2^ and 11.15 mW cm^−2^, respectively, at a current density of 10 mA cm^−2^ and operating voltage of 2.25 V (considering the IR drop). The significant gain in the performance of SCs is due to excellent electroactive surface per unit area (surface roughness 97.6 nm) of GPU composite and high electrical conductivity (0.318 S cm^−1^). The fabricated SCs show stable response for more than 15 000 charging/discharging cycles at current densities of 10 mA cm^−2^ and operating voltage of 2.5 V (without considering the IR drop). The developed SCs are tested as energy storage devices for wide applications, namely: a) solar‐powered energy‐packs to operate 84 light‐emitting diodes (LEDs) for more than a minute and to drive the actuators of a prosthetic limb; b) powering high‐torque motors; and c) wristband for wearable sensors.

## 1 Introduction

Electrical energy holds the key for advances in several emerging fields which include wearable systems,^1^, ^2^, ^3^, ^4^ robotics,^5^, ^6^ and autonomous vehicles.^7^ As a result, significant progress has been made in energy harvesting,^8^, ^9^, ^10^ low‐power electronics,^5^, ^11^ and numerous electrochemical based energy storage technologies have been explored meet the demand in these applications.^7^, ^12^, ^13^, ^14^, ^15^, ^16^, ^17^ Currently, the electrochemical based energy storage is largely based on Li ions batteries (LIBs), sodium ion batteries, or zinc–air batteries.^13^, ^14^, ^15^, ^18^, ^19^, ^20^, ^21^ In particular, LiBs offer high energy density (≈500 W h kg^−1^) and benefit from well‐developed manufacturing processes.^14^, ^18^, ^19^ But majority of them are not flexible and their weight, low power density, long charging time (1–2 h), heat generation,^22^ environmental concerns,^18^, ^19^, ^22^, ^23^ etc. limit their use in applications such as wearable systems. These limitations have already caught the attention of the research community, as evident from various works on flexible/stretchable batteries^12^, ^13^, ^24^, ^25^ and supercapacitors (SCs) with long cycling stability.^26^, ^27^, ^28^ In particular, the SCs offer excellent energy and power densities with low‐cost of fabrication.^26^, ^27^, ^28^, ^29^, ^30^, ^31^ Further they offer rapid charging (minutes vs hours in LiBs), long life cycle, do not generate heat^26^, ^30^ and are generally environment friendly.^28^, ^29^ With flexible and stretchable form factors, SCs can also conform to curved surfaces.^28^ Among various types of SCs, the electrochemical double layer capacitors (EDLCs)^28^, ^29^ based on carbon materials are the most promising^28^, ^29^, ^30^, ^31^, ^32^ because of their long lifetime (more than 10^6^ charging/discharging cycles), low environmental impact, ease of maintenance, and flexible form factors.^26^, ^27^, ^30^

The performance of SCs is mainly governed by their structure, surface morphology, electrolytes, and the electrochemical and electrical properties of active electrodes.^26^, ^30^, ^33^, ^34^, ^35^ For this reason, the choice of electrode materials and the electrolyte are critical. Since the energy storage (areal energy density, *E*_A_ = *C*_A_ *V* ^2^/2) depends on the potential window (*V*) and the specific capacitance (e.g., areal capacitance *C*_A_), researchers have focused on the ways to improve these values.^35^, ^36^, ^37^, ^38^ For example, a variety of carbon‐based structures (e.g., graphene foam,^35^ reduced graphene oxide (rGO),^36^ etc.) have been explored for EDLC fabrication. The choice of electrolyte is also important to increase the V and hence the energy density.^37^ Table S1 in the Supporting Information provides a comparison of *C*_A_, and *V* for EDLCs developed with various active carbon‐based materials. The low values of *C*_A_ (<10 mF cm^−2^) reported in the majority of the SCs can be attributed to the lack of electroactive surface per unit area needed to store the charge at the electrode–electrolyte interface.^33^, ^38^ The electrodes with multilayer structures have been explored to overcome such issues and improve the value of *C*_A_.^38^ For example, a 3D‐graphene/graphite‐paper based SC showed energy and power densities of 1.24 µWh cm^−2^ and 25 µW cm^−2^, respectively.^38^

**Figure 1:**
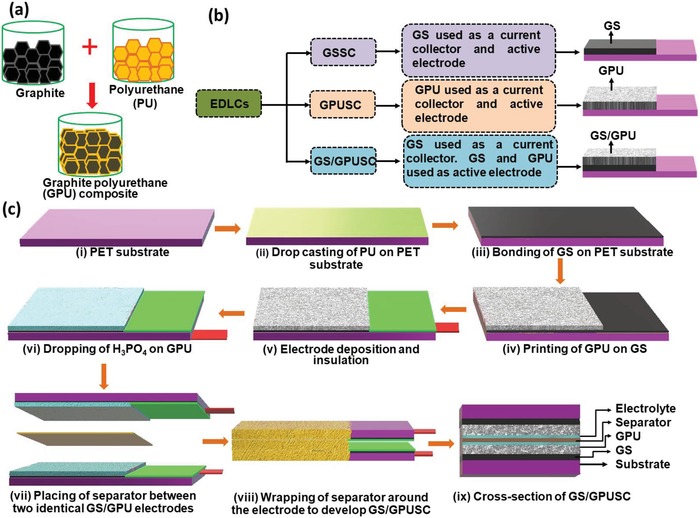
a) Steps for preparing GPU resin composite. b) Block diagram showing the fabricated EDLCs. c) i–vii) Schematic illustrations of the fabrication steps for GS/GPUSC and viii,ix) the cross‐sectional view of the resulting SC.

Here we present new graphite–polyurethane (GPU) composite (1:1 wt%) based electrodes for SC fabrication (**Figure** **1**a). The SCs with GPU composite and layered graphene sheet (GS) demonstrate a stable response with a potential window of 2.25 V, an energy density of 10.22 µW h cm^−2^, a power density of 11.15 mW cm^−2^, and long‐life times above 15 000 charging/discharging cycles (measured at 2.5 V). For the purpose of evaluation, three type of SCs were fabricated. These are: i) GS based SC, namely GSSC; ii) GPU based SC, namely GPUSC; and iii) layered GS and GPU composite‐based SC, namely GS/GPUSC (Figure 1b). Among these, the GS/GPUSCs exhibit best performance due to the use of conductive GPU composite electrode (Figure 1a,b), which offers increased electroactive surface per unit area for active ion exchange and charge transfer. The layer‐on‐layer assembly of GS and GPU resin composite also helps, as it lowers the resistance and increases the effective capacitance (Figure 1c). Furthermore, the polyester/cellulose blend separator filled with H_3_PO_4_ electrolyte is physically wrapped around the electrode to increase the wettability of the electrode–electrolyte interface (Figure 1c). The performance of the devices (GSSC, GPUSC, and GS/GPUSC) was evaluated by means of a thorough structural, morphological and electrochemical characterization of both the electrodes and the resulting SC. The fabricated SCs were also evaluated under different static and dynamic bending conditions. Finally, the wider applicability of fabricated SCs is demonstrated by implementing: a) solar‐powered energy‐packs to operate 84 light‐emitting diodes (LEDs) for more than a minute and to drive the actuators of a prosthetic limb; b) powering high‐torque motors; and c) wristband for wearable sensors.

## 2 Results and Discussion

### 2.1 Morphological and Structural Characterization

**Figure 2:**
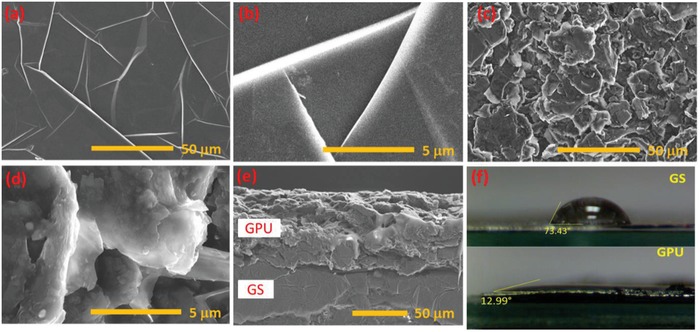
SEM images of a,b) GS and c,d) GPU films. e) Cross‐sectional view of GPU/GS heterostructure. f) Contact‐angle measurements of GS (top) and GPU films (bottom).

Surface morphology of GS (**Figure** **2**a,b) and GPU (Figure 2c,d) films have been characterized by scanning electron microscopy (SEM). SEM results reveal that the morphology of GPU composite is rough, exhibiting a layered structure consisting of graphite microflakes (Figure 2c,d). In contrast, GS morphology (Figure 2a,b) shows a polycrystalline (wrinkle) topography, with less surface roughness and less defined grain boundaries. In nanostructured materials, the nanometric thickness of the material can drastically affect the mechanisms governing the charge transport through GS and hence it acts as an excellent current collector in this SC. The compactness of the resulting structure in GPU was attributed to the polyurethane (PU) matrix (Figure 2d), promoting the adhesion and strong interaction between graphite flakes, as shown in the cross‐section views of GS and GPU film in Figure 2e. This morphological property is partially responsible for the high conductivity of the electrodes, as explained later. In addition to this, the difference in the surface morphology plays a major role in the roughness of the film and it leads to electrolyte interaction of the film. The surface wettability of both GS and GPU films has been characterized by means of contact‐angle measurements (Figure 2f). Aqueous H_3_PO_4_ electrolyte was drop casted on top of both GS and GPU film surface to study the contact angle of the resulting droplet (θ). Contact‐angle measurements reveal that GS film is more hydrophobic (θ = 73°) than the GPU film (θ = 13°), as presented in Figure 2f. In this regard, the GPU film prevents the issue arising from greater hydrophobicity component, i.e., the nanostructured and rough surface of these films offer better interaction surface for the electrolyte. The less hydrophobicity due to the surface roughness is required for high performance of SCs and was observed in the GPU electrode.

The surface roughness of GS and GPU films has been also analyzed by using atomic force microscopy (AFM) (Figure S1, Supporting Information). Figure S1a,b in the Supporting Information presents 3D AFM images of GS and GPU films, respectively. From those figures, one can clearly observe that GPU shows higher surface roughness than GS. AFM analysis shows that GS and GPU films have an average surface roughness (*R*_a_) of 0.2 and 97.6 nm, respectively.

**Figure 3:**
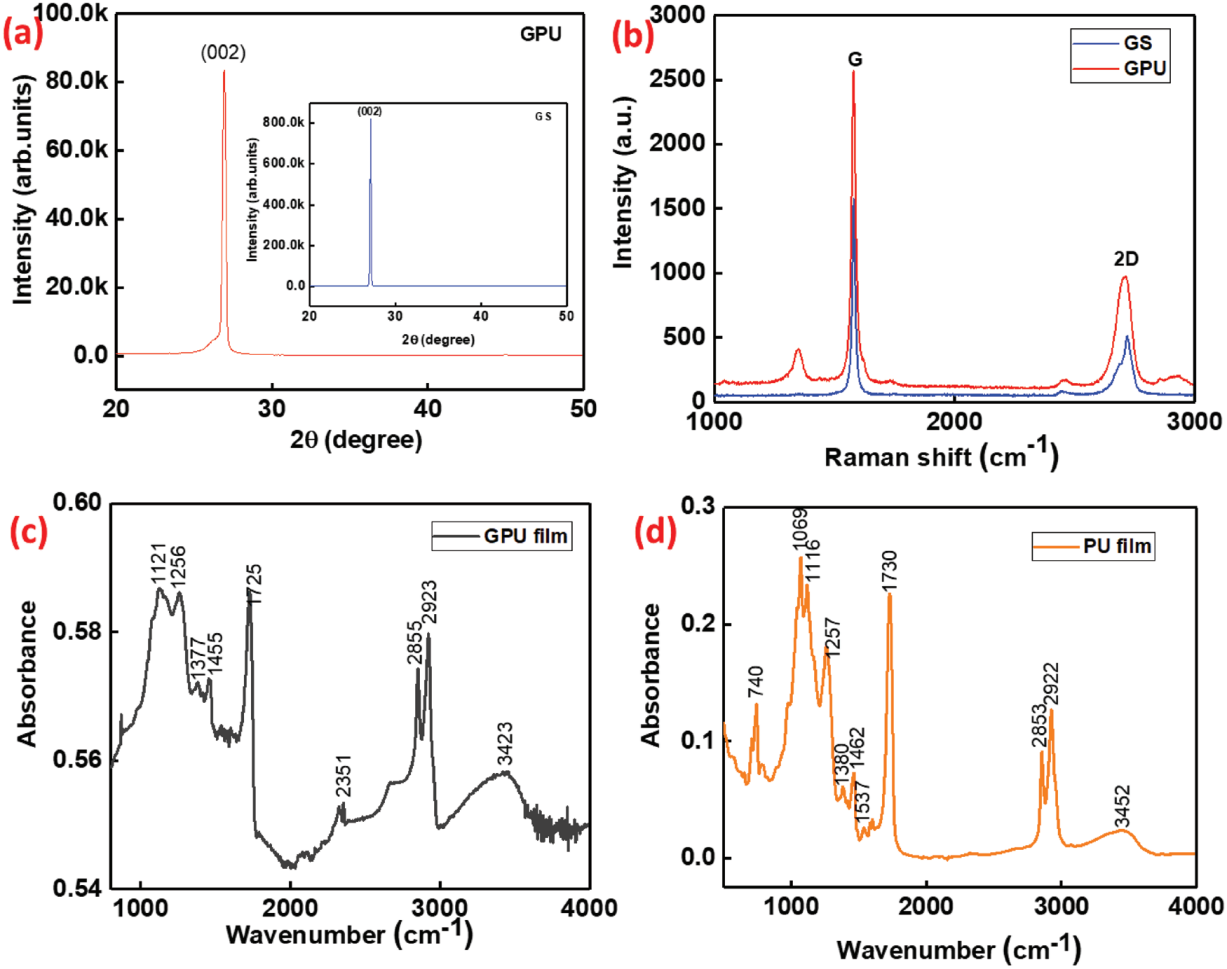
a) XRD pattern of GPU and GS film (inset). b) Raman spectra of GS and GPU films. FTIR spectra of c) GPU and d) PU films.

The crystalline structure of the GPU and GS films has been analyzed by X‐ray diffraction (XRD) (**Figure** **3**a). XRD pattern taken from as‐prepared GPU films indicates that crystal grains are highly oriented along (002) direction (2θ = 26.8°). The sharp and intense peak at 2θ = 26.8° corresponds to the diffraction peak of graphite at (002). To further confirm the crystalline orientation of the GPU, the structure was compared with XRD of GS as shown in inset of Figure 3a. It was found that the peak for both GS and GPU is oriented in the same direction and it reveals the crystalline structure of the GPU composite.

Raman spectroscopy was used to investigate the crystalline property of the GPU films in comparison with GS. The analysis shows two major peaks at 1570 and 2717 cm^−1^ for GS, which are identified as G and 2D bands of graphene (Figure 3b).^39^ The lack of D peak in the Raman spectrum of GS film confirms its high crystal quality. On the other hand, GPU Raman spectrum (Figure 3b) exhibits a small D peak centered around 1345 cm^−1^, arising from the existence of structural defects.^39^ The ratio between D and G peak intensities (*I*_D_/*I*_G_) resulted in 0.16, confirming the formation of the small graphitic domains in the structure.^39^ Comparing 2D bands of GPU and GS films (Figure 3b), an energy shift from 2716 cm^−1^ (GS) to 2708 cm^−1^ (GPU), i.e., around 8 cm^−1^, is observed. The origin of this shift is caused by the influence of the PU resin in the heterostructure composites.^39^

The functional groups present in the GPU film were investigated by using Fourier transform infrared (FTIR) spectroscopy and compared to those obtained in pure PU films. Among the absorption peaks, the broad and less intense peaks observed at 3452 and 3423 cm^−1^ can be attributed to the —OH stretching of polyols in the PU. The stretching vibration of —CH_3_ and —CH_2_— is represented in the absorption peaks observed at 2922 and 2853 cm^−1^, respectively, for both PU and GPU films (a slight shift in peak position is observed between them) in Figure 3c,d. The absorption peaks observed at 1730 and 1725 cm^−1^ corresponds to ester‐based C=O stretching. The stretching vibration of amine (C—N) functional group is represented by the medium peak at 1256 cm^−1^ for GPU (Figure 3c) and 1257 cm^−1^ for PU film (Figure 3d). The peaks at 1121 and 1069 cm^−1^ correspond to the polyester's C—O group.^39^, ^40^ The presence of amine and hydroxyl group shows that more ions can be attached into the lattice of the graphite‐PU matrix,^40^ which further enhance the performance of the SC, as it will be demonstrated later.

### 2.2 Working Mechanism

**Figure 4:**
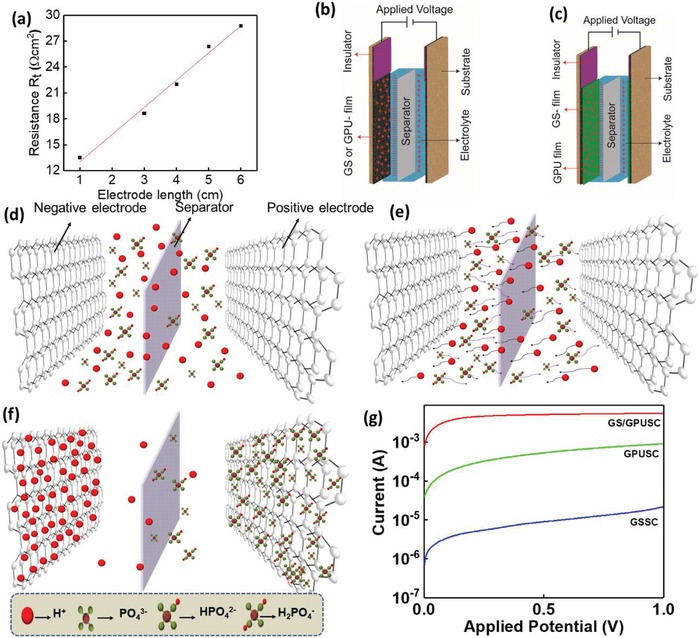
a) Total resistance versus length of the electrode; calculation of the electrode conductivity by transfer length method. 3D schema of EDL for b) GSSC and c) GPUSC. 3D schema describing the formation of EDL and charging mechanism for GSSC, GPUSC, and GS/GPUSC, comprising: d) ions distribution before applying potential, e) ion absorption while charging the SC electrodes, and f) positive and negative ions diffusion from H_3_PO_4_ electrolytes to the electrodes of SC after charged. g) *I–V* curves of GSSC, GPUSC, and GS/GPUSC measured during charging.

In EDLCs, an electric double layer (edl) is formed at the interface between the conductive electrode and the electrolyte, enabling the effective storage of energy. High electrical conductivity of the electrode is expected for the high performance of EDLC. The electrical conductivity of both GPU and GS electrodes was measured using a transfer length method. The net resistance (*R*_t_) per unit area of GPU was obtained by *R*_t_ = *R W t*, where *R* is the resistance of GPU, and *W* and *t* are the width and thickness of the electrode, respectively. Representing *R*_t_ as a function of length of the electrode (*L)* (**Figure** **4**a), (resistivity (ρ) measured from the slope of the Figure 4a). The conductivity of the electrode can be calculated from the slope (0.318 S cm^−1^). The multilayered GS shows a high conductivity of 126 S cm^−1^, indicating high crystallinity and surface morphology of the GS (Figures 2 and 3).

The edl formation depends on factors such as surface properties of electrode (surface area, porosity, roughness, etc.) electrolyte behavior, separator, and distance between electrodes.^26^, ^33^, ^41^, ^42^ To study the edl of SCs, three types of EDLCs have been characterized, including GSSC, GPUSC (Figure 4b), and GS/GPUSC based on layer‐by‐layer configuration of electrodes (Figure 4c). The three stages of the charging process for SCs is shown in Figure 4d–f and is common for all three SCs. In the absence of external applied potential, cations (H^+^) and anions (PO_4_^3−^, HPO_4_^2−^, and H_2_PO^−^)^37^, ^41^ from H_3_PO_4_ are randomly distributed throughout the electrolyte volume (Figure 4d). When a potential is applied between electrodes, both cations and anions from the electrolyte are driven toward the cathode and anode, respectively, diffusing across the separator (Figure 4e), and finally, being absorbed along the electrodes surface (Figure 4f). As these ions get accumulated at the surface of the electrodes, the edl is formed at each electrode surface (Figure 4f).^41^, ^42^ For GSSC, the initial measured discharge current (after charging to 1 V) is around 22 µA and for GPUSC it is 0.91 mA, which is almost 42 times higher than GSSC. In comparison with GSSC and GPUSC, the GS/GPUSC shows the highest acquired current (in the range of 5.5 mA). Figure 4g presents a comparison of measured current in these types of SCs. The observed differences in current thresholds with the same potential (1 V) allowed us to understand the influence of electrode morphology and configuration on the electrochemical and capacitive performance of the devices.

### 2.3 Electrochemical Performance

**Figure 5:**
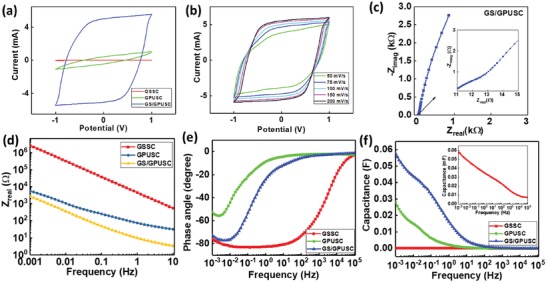
a) Comparison of CV curves of the GSSC, GPUSC, and GS/GPUSC at 100 mV s^−1^. b) CV curves of the GS/GPUSC at different scan rates. c) Nyquist's plot of GS/GPUSC at frequencies ranged between 1 mHz and 100 kHz; insets: plots for high frequencies range. g) Comparison of *Z*_real_ versus frequency (1 mHz to 10 Hz) for GSSC, GPUSC, and GS/GPUSC devices. h) Bode angle plot for GSSC, GPUSC, and GS/GPUSC. i) Capacitance variation versus frequency for GSSC, GPUSC, and GS/GPUSC; inset: plot for GSSC.

The high electroactive surface area of the GPU composite electrode (similar to other graphite–polymer composites^43^, ^44^) generates higher current per unit area than the pure graphene conductive electrode in aqueous solutions. For the sake of comparison, cyclic voltammetry (CV) analysis was carried out at 100 mV s^−1^ in all types of SCs fabricated in this work, i.e., GSSC, GPUSC, and GS/GPUSC (**Figure** **5**a). The quasi‐rectangular shape of all the CV curves indicates an EDLC mechanism for fabricated SCs.^44^ Even though CV curve for GSSC shows rectangular shape, the observed current is in µA range, but for GPUSC in the range of 1 mA (Figure S2a,b, Supporting Information). Figure 5b represents the CV profile of GS/GPUSC over scan rates ranged between 50 and 200 mV s^−1^. The CV profile at 200 mV s^−1^ indicates a fast response time and excellent charge storage capability of the fabricated GS/GPUSC device. Moreover, it is worth noticing that the increase of the scan rate leads to the increase of the accumulated charges in GS/GPUSC (Figure S2c, Supporting Information). This indicates the domination of diffusion‐controlled reaction of the electrodes. From CV analysis, one can observe that the electrode configuration, and the new GPU composite proposed in this work, foster the attraction of a higher density of ions toward the surface of the electrode, thus enhancing the charge storage capacity of resulting SCs.^40^ Moreover, the CV analysis reveals that there is no strong redox reaction due to the influence of PU as compared to other conducting polymers or conductive Ag based epoxy, which were used as binder in the graphite paste.^35^ For the study of the ion exchange mechanism in SCs, the electrochemically active surface area (ECSA) is one of the key parameters to be investigated. For GS/GPUSC fabricated in this work, the ECSA was estimated from the capacitance of the EDL (*C*_dl_) and the specific capacitance (*C*_s_) by using the expression ECSA = *C*_dl_/*C*_s_. The value of *C*_dl_ was determined by measuring slope of the double‐layer charging from the scan‐rate dependence of CVs (shown in Figure S2d, Supporting Information). The *C*_s_ was measured from the CV analysis at 100 mV s^−1^. By using the above expression for ECSA, the active surface area for ion exchange is found to be 0.45 cm^2^. Furthermore, the separator also has a strong influence^45^ on the electrochemical property of the electrodes as described in the Supporting Information. The area, recorded current, and the shape of CV curve at 100 mV s^−1^ scan rate for GS/GPUSC are lower when single separator was used (shown in Figure S2e, Supporting Information) as compared to Figure 5a when separator is wrapped around the electrode.

The almost straight line observed (from electrochemical impedance spectroscopic (EIS) analysis) in the low‐frequency region of Nyquist plots obtained in GS/GPUSC (Figure 5c) implies the Warburg impedance due to the ion diffusion/transport from the electrolyte to the electrode surface. The Nyquist plots for GSSC and GPUSC are presented at Figure S2f,g, respectively in the Supporting Information. Due to the hydrophobic behavior of GS (Figure 2f) affecting the ion exchange mechanism, the resistance (Z_real_) of GSSC at low frequencies (1 mHz to 10 Hz), exhibits higher values (89 Ω to 0.67 MΩ) than GPUSC (428 Ω to 4.3 kΩ) and GS/GPUSC (16 to 849 Ω) (comparison plot is given in Figure 5d). This high resistance in the ionic exchange for GS leads to the storage of limited amount of charges, lowering the value of capacitance. However, the new GS/GPUSC offers a low resistance and hence high conductance for faster ionic exchange. The conductive electroactive GPU flakes have good interaction with the electrolyte due to the urethane groups in the hard domains of PU and the polyethylene glycol polytetramethylene ether glycol units in the soft domains of PU.^25^, ^40^, ^44^ This facilitates the ionic diffusion and hence wettability (low hydrophobicity) which leads to high conductance for ionic exchange. This reveals that, due to ionic and electronic transport, the resistance offered by electrodes in each device influences the electrochemical performance. In addition to this, the Nyquist plots for GSSC (inset of Figure S2f, Supporting Information) and GS/GPUSC (inset of Figure 5c) do not exhibit the semicircle like behavior observed at high frequencies in GPUSC (inset in Figure S2g, Supporting Information). This indicates that the interfacial charge transfer resistance of both devices is low due to the high conductivity of electrodes and that the stored charges are transferred to the external electrode. Moreover, the printing of GPU electrode layer on the top of highly conductive graphene sheet reduces the electron transfer resistance and further reduces the overall resistance of the GS/GPUSC based active electrode. In contrast, the small semicircle arc like trend observed at high‐frequencies for GPUSC devices (Figure S2g, Supporting Information) would indicate a relatively high resistance of the material while transferring the electron to an external conducting electrode. Further, the depressed semicircle in the Nyquist plot can be attributed to the rough surface of the GPU flakes. The resistance values of each device (*R*_s_) in the high‐frequency range are also different because of the influence of electrolyte and contact resistance of a material (comparison given in **Table** **1**). The GS/GPUSC exhibited *R*_s_ of 11 Ω at high frequencies, mainly due to the high conductivity and layer‐by‐layer configuration of the electrode. The smaller value of *R*_s_ also provides better contact between electrodes of SC and the electrolyte, thereby contributing to the improvement in the capacitive performance of the resultant SC.^33^, ^45^

**Table 1:** Comparison of electrochemical performance of fabricated SCs

SC	Resistance (*Z*_real_) (at 10 Hz–1 mHz)	Solution resistance *R*_s_ [Ω]	Phase angle [degree]	Capacitance at 1 mHz
GSSC	89 Ω to 0.67 MΩ	4.55	−83	0.59 µF
GPUSC	428 Ω to 4.3 kΩ	334.68	−55	30 mF
GS/GPUSC	16 Ω to 849 Ω	11.06	−77	60 mF

The capacitive performance of the device was investigated with Bode plot of EIS analysis.^33^, ^35^, ^46^ Figure 5e reveals the variation of phase angle for GSSC, GPUSC, and GS/GPUSC. From this analysis, it was found that at low‐frequencies, the phase angles for GSSC and GS/GPUSC reach the maximum values of −83° and −77°, respectively, confirming the capacitive behavior of the SC. At this low range of frequencies, the Bode impedance (Figure S2h, Supporting Information) decreases by increasing the frequency, revealing an ideal capacitive nature and good stability of the SC under analysis. The magnitude of capacitance value depends on the performance of ionic exchange and the area of active electrode that is accessible for charge accumulation. We observed that at the low‐frequency range, the GS/GPUSC shows a high capacitance, shown in Figure 5f. For example, at 1 mHz, the capacitance of GSSC, GPUSC, and GS/GPUSC are 0.59 µF (see inset of Figure 5f), 30 and 60 mF, respectively. This result can be explained due to the deep diffusion of ions from H_3_PO_4_ electrolyte inside the pores of GS/GPUSC material, accessing more electrode surface, and therefore, contributing to obtain a higher capacitance value at low frequencies.^21^ Hence, the EIS analysis reveals that the new electrode configuration of GS/GPUSC leads to a significant enhancement of the electrochemical and supercapacitance performance of the device. However, when using a single separator to fabricate the SC, the electrochemical performance of the device is strongly influenced (see Figure S2i,j, Supporting Information).

### 2.4 Charge–Discharge Analysis

**Figure 6:**
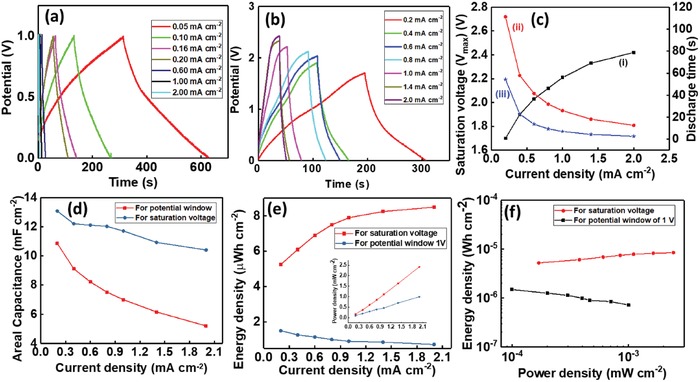
GCD curves for GS/GPUSC at different current densities in H_3_PO_4_ electrolytes for a) the potential window of 0–1 V and b) maximum saturation voltage. c) the variation of i) *V*_sat_, ii) discharge time for *V*_sat_, and iii) discharge time for potential window of 0–1 V with current density for GS/GPUSC. d) Comparison of areal capacitance with current density at 1 V and *V*_sat_ for GS/GPUSC. e) Relationship between areal energy density and power density (inset) with current density for *V*_sat_ and for 0–1 V potential. f) Comparison of Ragone plot of energy density versus power density for *V*_sat_ and potential window of 0–1 V.

As observed from CV and EIS analysis, the electrochemical performance of GS/GPUSC is better than GSSC and GPUSC. This was also confirmed by the Galvanostatic constant current charge–discharge (GCD) measurement (Figure S3a–c, Supporting Information).In the case of GSSC, the charging–discharging occurred at very low current densities (in the range of 2–8 µA cm^−2^), as shown in Figure S3a in the Supporting Information. Due to an improved surface‐active area for charge storage, the charging of the GPUSC requires higher current densities than GSSC (Figure S3b, Supporting Information). For GPUSC, the stored charges get discharged at a slower rate (Figure S3b, Supporting Information) as compared to GSSC. However, at low current density (0.05 mA cm^−2^), the discharge time of GS/GPUSC is approximately ten times higher than GPUSC device, as shown in Figure S3c in the Supporting Information. This means the lack of charge storing capability or low electroactive surface area for ion exchange results in quick discharge in the graphene film. It is already reported that the highly conductive graphene based SCs have poor electrochemical and supercapacitance performance.^38^ This is because of pure graphene film‐based electrodes usually suffer from aggregation and/or restacking due to the strong π–π* interactions between the graphene oxide sheet.^38^, ^45^ Moreover, the GS is hydrophobic in electrolyte‐based environments (Figure 2f). On the other hand, the GPU surface provides greater electroactive surface per unit area (less hydrophobicity, Figure 2f) for more current, as shown in Figures 4g and 5b (for different CV scan rate) and this leads to improved charge storage capability. The ions accumulated on the edl from the electrolyte, eventually diffuse into the bulk of the GPU material. Due to low resistance of the electrode and particular structure, ions are rapidly and efficiently transferred through GPU channel to the GS current collector, overall, benefiting the energy storage mechanism. The GPU network composite also suppresses the aggregation or restacking due to the strong π–π* interactions in graphene oxide. Finally, the layered structure of the GS/GPUSC electrode, leading to lower values of contact and diffusion resistances—as confirmed by EIS analysis (shown in Table 1) helped to reduce the potential energy loss in the SC. Due to this excellent electrochemical performance, the GS/GPUSC exhibits good charging–discharging cyclic stability in both the low and high current densities regimes, as shown in **Figure** **6**a, for potential range of 0–1 V).

The saturation voltage (*V*_sat_) that indicates the maximum potential window of an SC is a key parameter to be maximized in order to enhance the energy or power storage capability.^33^, ^45^ We have measured the value of *V*_sat_ by applying a constant current to the SC and measuring the voltage accumulated in the SC. The *V*_sat_ is obtained under a tolerance of 10%, which means, two consecutive voltage measurements showing a difference below 10% (Δ*V* = 100 * (*V_i_*_+1_ – *V_i_*)/(*V_i_*_+1_ + *V_i_*) ≤ 10, where *i* = 1, 2, 3, …). In this scenario, we have considered that the SC cell reached the saturation state. After reaching the saturation potential (fully charged), the SC starts to discharge. We observed that *V*_sat_ varies with the applied current densities due to variations in the amount of charge stored, as shown in Figure 6b for GS/GPUSC. The performance of GSSC (Figure S3d, Supporting Information) and GPUSC (Figure S3e, Supporting Information) at *V*_sat_ is discussed in the Supporting Information. The cell configuration and solvent in the electrolytes are the two major factors which affect the value of *V*_sat_ and hence the performances of SC.^45^ The majority of EDLCs reported with aqueous acidic or basic electrolytes (e.g., NaOH, H_3_PO_4_, H_2_SO_4_, NaCl, etc.) have the maximum operating voltage of 1.3 V (shown in Table S1, Supporting Information). In this work, even with aqueous H_3_PO_4_ as an electrolyte, we were able to achieve the maximum operating voltage of 2.42 V at a current density of 2 mA cm^−2^. The results presented so far, comprising morphological, structural, electrochemical, and super‐capacitive characterization, have allowed us to attribute this drastic enhancement of the *V*_sat_, to the properties of electrodes and the cell configuration. In this regard, the GPU is found to be operable at a higher potential window in aqueous electrolyte (H_3_PO_4_ 80% concentrated), without chemical breakdown of the electrolyte.^25^, ^40^, ^43^, ^44^ In the aqueous electrolytes the water generally decomposes or breaks down at 1.229 V. In our previous work, we observed such a breakdown, and as a result a low voltage performance was observed for graphene‐Ag epoxy‐graphene foam‐based SCs.^35^ However, the breakdown voltage is also influenced by the electrodes used in SCs as may be noted from other works such as graphite–PU composite based devices with aqueous electrolyte and operating in high potential window of 1.75 V.^43^ Our electrochemical studies show similar behavior for GPU. The electrolyte‐electrode is found to have very good interaction and low contact resistance (11 Ω) without any charge transfer resistance. Similarly, we did not observe any significant redox peaks due to the electrode reaction. This means the electrode material is not degrading during the electrochemical reaction. The *V*_sat_ for GS/GPUSC is also higher as compared to GPUSC based SC. Hence, the results show that the layered configuration of the electrodes enables the SC to operate in a high potential window. The high conductivity of the electrodes (0.38 S cm^−1^ for top (GPU) and 126 S cm^−1^ for multilayered GS (bottom)) also influences on these performances.

To obtain the saturation potential during the measurement we maintained long time in charging in the *V*_sat_ region and this led to the GCD curve having an asymmetric shape (Figure 6b and Figure S3d,e, Supporting Information). It is worth noticing that even though the GSSC and GPUSC can reach high *V*_sat_ (Figure S3d,e, Supporting Information), both SCs discharge at a faster rate than GS/GPUSC at high current densities. As a result, the applicability of GSSC and GPUSC will be limited. The poor operation at high current density and the quick discharging of these devices can be overcome by using the double‐layered structure as in the case of GS/GPUSCs. The self‐discharge of this SC/GPUSC under *V*_sat_ condition is shown in Figure S3f in the Supporting Information. In addition, to evaluate the influence of electrolyte on the performance of the SCs, we have carried out GCD measurements for GS/GPUSC with NaOH electrolyte and compared with results obtained by using H_3_PO_4_ electrolyte (Figure S3g,h, Supporting Information). We noted that, as compared to H_3_PO_4_, the NaOH shows faster discharging times, which could be due to low ionic concentration for edl formation. The H^+^ ions in H_3_PO_4_ can diffuse more efficiently into GPU matrix than Na^+^ ions from NaOH electrolyte.^41^

In this work, we observed that the Coulombic efficiencies, which depends on the charging conditions of the SCs. To evaluate the Coulombic efficiency, we measured the charging and discharging time for both the fixed potential window and for the saturation potential. In the case of fixed potential window for GS/GPUSC, the charging and discharging time are almost same (314 s) (Figure S4a, Supporting Information) at current density of 0.5 mA cm^−2^ and this means the Coulombic efficiency is close to 100%. However, for the saturation potential we noted that the SC took long charging time particularly at a low current density to store the maximum charge. We observed that *V*_sat_ varies with the applied current densities due to variations in the amount of charge stored. The charging and discharging time of the *V*_sat_ measurement is different and as a result the Coulombic efficiency of the device is 58%, shown in Figure S4b in the Supporting Information. After finding the *V*_sat_ value, we applied high current density to reach the maximum potential window. The charging and discharging time for this measurement (Figure S4c, Supporting Information) shows the Coulombic efficiency of 69.4%. Hence these measurement reveals that the Coulombic efficiency of the device depends on the charging conditions.

### 2.5 Operational Efficiency of Graphite–Polyurethane SC

From electrochemical analysis (both CV and EIS) and GCD analysis, we observed that the GS/GPUSC shows best performance. Following this, a detailed study of GS/GPUSC was carried out to evaluate their operational efficiency. The expressions used for operation efficiency calculation are given in the Supporting Information. To measure the performance of SCs, we used both 1 V (a fixed potential) and *V*_sat_ with different current densities. From Figure 6c and curve (i) we can conclude that by increasing current density (in the range of 0.2–2 mA cm^−2^) the *V*_sat_ increases. Further, Figure 6c shows the measured discharging time of GS/GPUSC for operating potentials of *V*_sat_ (curve (ii)) and 1 V (curve (iii)) at different current densities. By using these potential values and time we measured the operational efficiently of the SCs. The GS/GPUSC exhibited a high value of areal capacitance (*C*_A_) (15.79 mF cm^−2^) at a low current density of 0.05 mA cm^−2^ for 1 V potential window. Figure 6d compares the variation of *C*_A_ for GS/GPUSC device operated in a potential of 1 V and *V*_sat_ for the current density variation in the range of 0.2–2 mA cm^−2^. Due to the longer discharging time the obtained value for *C*_A_ was higher in the case of *V*_sat_. In fact, the values of *C*_A_ obtained with this GS/GPUSC are higher than or comparable with most of the reported carbon‐based SC, as shown in Table S1 in the Supporting Information. By comparing the performance obtained in GSSC, GPUSC, and GS/GPUSC, we noted that GS/GPUSC presents the best operational efficiency as recorded in **Table** **2**. Table S2 in the Supporting Information shows the operational efficiency for GS/GPUSC in NaOH electrolyte. The observed *C*_A_ for NaOH based GS/GPUSC is 1.13 mF cm^−2^ in a potential window of 1 V and is comparatively lower value for H_3_PO_4_ electrolyte‐based SC.

**Table 2:** Comparison of performances of GSSC, GPUSC, and GS/GPUSC

SC	Current density	Areal capacitance	Energy density	Power density
GSSC	2 µA cm^−2^	10.04 µF cm^−2^	1.4 nW h cm^−2^	1 µW cm^−2^
GPUSC	0.05 mA cm^−2^	1.15 mF cm^−2^	0.21 µW h cm^−2^	0.024 mW cm^−2^
GS/GPUSC	0.05 mA cm^−2^	15.79 mF cm^−2^	2.21 µW h cm^−2^	0.025 mW cm^−2^

Here, we have observed two types of characteristics for areal energy density (*E*_A_) while varying the current density. For low current density (0.2 mA cm^−2^) the energy and power densities of GS/GPUSC are 5.25 µW h cm^−2^ and 0.17 mW cm^−2^ in the case of *V*_sat_. For potential window of 1 V, these values are 1.5 µW h cm^−2^ and 0.10 mW cm^−2^, respectively. For a high current density (2 mA cm^−2^) the energy and power densities are 8.50 µW h cm^−2^ and 2.43 mW cm^−2^, respectively, in the case of *V*_sat_ and 0.72 µW h cm^−2^ and 1.0 mW cm^−2^, respectively, for potential window of 1 V. Thus, the energy density corresponding to *V*_sat_ increases with the current density. However, for fixed potential window (1 V) the energy density decreases with the increase in the current density, as shown in Figure 6e. For both saturation and potential windows, the power density increases with the current density, as shown in the inset of Figure 6e. The overall performance of the GS/GPUSC is shown in a Ragone plot in Figure 6f, which shows that the device working in saturation voltage exhibits an increase in energy density with increase in the power density (*P*_A_). This is contrary to most of the reported works, which show decrease in energy density with power density because of their limited operating voltage for all current densities.^30^, ^33^, ^38^ For a potential window of 1 V we also noted the conventional behavior from our SCs, also as shown in Figure 6f (in Black). Hence, we found that, by increasing the current density, *V*_sat_ and the corresponding *E*_A_ also increase. It was also noticed that at high current density the SC shows IR drop in voltage, which affects the Coulombic efficiency of the device. This IR drop has been considered in the calculations related to the energy and power densities. A further analysis was carried out to determine the performance of GS/GPUSCs under high current densities for high energy density applications (e.g., in motor).

**Figure 7:**
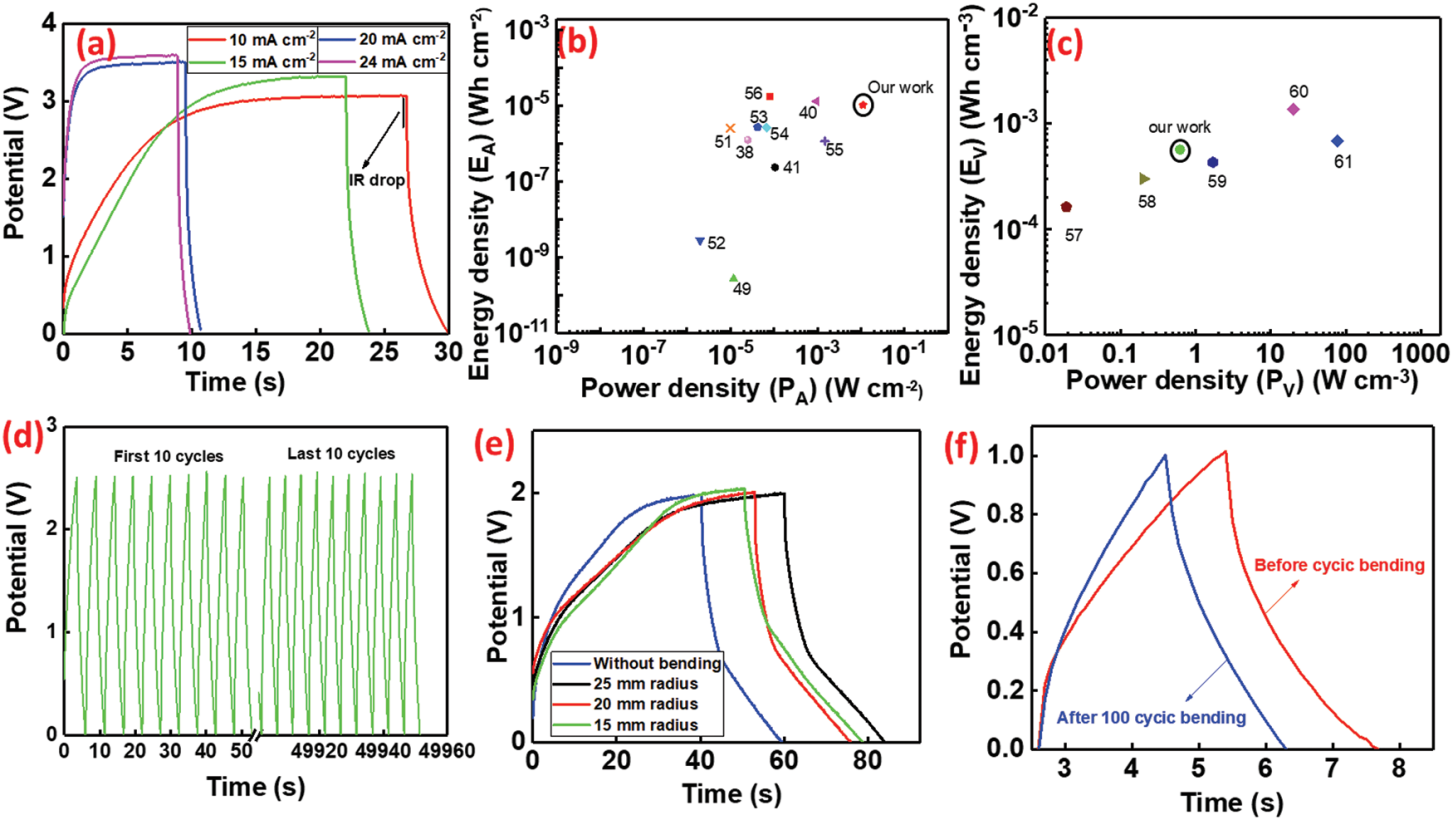
a) Performance of GS/GPUSC obtained at high current densities. Ragone plot comparing b) areal and c) volumetric energy density versus power density obtained in GS/GPUSC and in state‐of‐the‐art SCs. d) First and last ten cycles among 10 000 charging/discharging cycles of the GS/GPUSC. e) GCD curve under different bending radius. f) GCD curve before and after 100 bending cycles.

From this study, we have observed that the *E*_A_ increases with the current density up to a maximum value and then decreases due to IR drop (**Figure** **7**a). As similar to Figure S3d,e in the Supporting Information the GCD curve is not asymmetric shape due to long charging in saturation potential. The variation of *E*_A_ over a current density ranged between 0.2 and 24 mA cm^−2^ is shown in Figure S5a in the Supporting Information. While calculating the *E*_A_ and *P*_A_ of GS/GPUSC, we have considered such IR drop (equations given in the Supporting Information). The maximum energy density of GS/GPUSC was 10.22 µW h cm^−2^ at current density of 10 mA cm^−2^ with an operating potential of 2.25 V after IR drop (the value of *C*_A_ for this voltage is 14.79 mF cm^−2^). The value of power density in these conditions was estimated around 11.15 mW cm^−2^. The corresponding volumetric capacitance, energy, and power density of this devices are 0.822 F cm^−3^, 0.567 mWh cm^−3^, and 618 mW cm^−3^, respectively. For high power applications, GS/GPUSC can deliver a power density up to 22.63 mW cm^−2^ (at the current density of 24 mA cm^−2^) corresponding to an energy density of 6.2 µW h cm^−2^. The obtained *E*_A_ and *P*_A_ (10.22 µW h cm^−2^ and 11.15 mW cm^−2^) of the GS/GPUSC is much higher than the reported values for flexible and wearable EDLC based SCs (comparison is shown in Table S3, Supporting Information). For example, 1.24 µW h cm^−2^ at 25 µW cm^−2^ for 3D‐graphene/graphite‐paper,^38^ 3.84 µW h cm^−2^ at 0.02 mW cm^−2^ for carbon nanotube (CNT)/graphene wet‐spun fibers,^47^ 2 µW h cm^−2^ for graphene cellulose paper,^48^ 0.27 nW h cm^−2^ at 11.77 µW cm^−2^ for wrinkled graphene films,^49^ etc. As shown in Figure 7b, the results of our GS/GPUSC are comparable to the values reported based on graphene and conducting polymer composites (e.g., 12.9 µW h cm^−2^ at 954.3 µW cm^−2^ for PPy/GO^50^ and 2.52 µW h cm^−2^ at 0.01 µW cm^−2^ for polyaniline (PANI)/GO^51^). Further, the comparison of the volumetric energy and power densities obtained in our GS/GPUSC with other state‐of‐the‐art SCs is shown in the Ragone plot of Figure 7c. The observed results show that the fabricated SC shows excellent performance with higher volumetric power density as compared to the reported works of similar aqueous electrolyte‐based devices.

One of the major advantages of the fabricated GS/GPUSC is the cyclic stability of the cell for a long period of measurement at a high potential window. Even though some of the carbon based SCs reported in the literature have demonstrated long lifecycle stability, their performance was characterized at low potential windows (≤1 V) and at low current densities, hindering their applicability. In this work, we have demonstrated that the fabricated GS/GPUSC can operate in potential window up to 2.5 V, at high currents up to 50 mA, and for more than 15 000 cycles. For this initially 10 000 cycles were carried out. Figure 7d shows that the GCD measurement of a GS/GPUSC for initial and final ten cycles from 10 000 cycles. The capacitance of the SC for first cycle was 12.12 mF cm^−2^ and after 10 000 cycles it is 11.5 mF cm^−2^ (considering the IR drop in the measurement). Figure S5b in the Supporting Information shows that the GCD measurement of a GS/GPUSC for first and last cycle from 10 000 cycles. From this, it was observed that the potential 2.5 V of the device for the initial and final charge–discharge cycle is almost same and its power density is in the average range of 10 mW cm^−2^ after IR drop. These results predict an excellent stability for the presented device. The performance of GS/GPUSC after 15 000 cycles (i.e., additional 5000 cycles after the first measurement of 10 000 cycles,) is shown in the Figure S5d and described in Table S4 in the Supporting Information.

For wearable applications, the GCD measurements were also obtained for GS/GPUSC under different bending conditions. The performance of GS/GPUSC, under bending radius of 15, 20, and 25 mm at an applied current of 1 mA cm^−2^ shown in Figure 7e, highlights no significant change in the performance of the SC under the bending (see **Table** **3**). In addition to this, the measurements were carried out also under dynamic cyclic bending, with a bending radius of 24 mm for 100 cycles (an image of bending setup is shown in Figure S5c, Supporting Information). The results shown in Figure 6f (Movie S1, Supporting Information) do not indicate any significant change in the performance of the SC. Almost same discharging time (≈2.3 s) of the SC before and after cyclic bending prove the stability of the device and their potential use in wearable systems. Further flexibility analysis was carried out for GS/GPUSC by putting the SC on the inner surface of a 3D printed cylinder and the GCD measurements (shown in Figure S6, Supporting Information) shows stable charging/discharging performance.

**Table 3:** Comparison of performances of GS/GPUSC with and without bending at 1 mA cm^−2^

GS/GPUSC	*V*_sat_ [V]	Discharge time [s]	Areal capacitance [mF cm^−2^]	Energy density [µW h cm^−2^]	Power density [mW cm^−2^]
Without bending	1.99	25.10	12.61	6.93	0.99
25 mm radius of curvature	1.99	24.00	12.06	6.63	0.99
20 mm radius of curvature	2.00	25.10	12.55	6.97	1.00
15 mm radius of curvature	2.00	28.30	14.15	7.86	1.00

### 2.6 Application of SC

**Figure 8:**
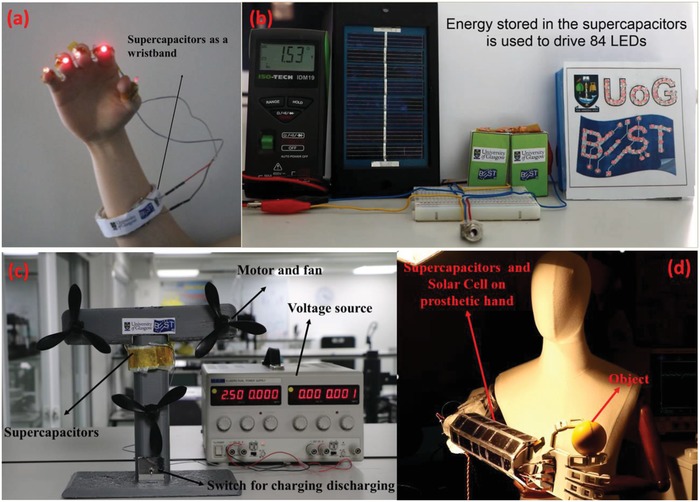
Application of flexible GS/GPUSC. a) Image of SC as a wristband. b) Self‐charging of the fabricated SC by solar cell and the stored energy used for operating 84 LEDs. c) SC used for operating a motor which is attached to a fan. d) SCs and solar cell wrapped around a prosthetic hand for operating the motors in fingers of the hand.

Stable and reliable operation of flexible SCs is crucial for several applications.^3^, ^5^ In this regard, we have demonstrated the applicability of fabricated GS/GPUSC to store and supply energy to different devices, including 1) a wearable wristband consisting of flexible SCs cells connected in series to power five LEDs (**Figure** **8** a) (GCD analysis presented in Figure S5e, Supporting Information); 2) for a self‐powered power pack system, two flexible SCs housed in a 3D printed package to act as an energy storage unit connected with a solar cell to power 84 LEDs (Figure 8b) for more than 1 min (Move 2, Supporting Information); 3) for a high energy density, SC powering a set of fans (Figure 8c). The performance of the SCs for single motor operation is shown in Movie S3 in the Supporting Information and three motors in Movie S4 in the Supporting Information. The developed SCs have also been used as an energy storage device in a solar‐powered prosthetic/robotic hand. The SCs serves as a buffer to offer higher current discharge during the operation of various motors of the prosthetic hand, which is not possible with solar cells alone. Together with solar cells, the SCs offer an attractive solution for future energy autonomous robotics and prosthetics (Figure 8d). In the Movie S5 in the Supporting Information, the index and thumb fingers are actuated through pulse width modulation (PWM) to perform grab and release action (block diagram and circuit shown in Figure S6, Supporting Information). The presented demonstration is the first time SCs and solar cells are used to power a prosthetic hand GS/GPUSC. The detailed discussion about the design of above‐mentioned applications of SCs including block diagrams are presented in the Supporting Information.

## 3 Conclusion

In conclusion, in this work we have reported a novel GPU composite‐based electrode for *EDL* SC applications. The high‐energy density SC has a layer‐by‐layer configuration, consisting of a graphene–graphite polyurethane resin composite. With excellent electroactive surface per unit area, the GPU composite exhibited excellent electrochemical and super‐capacitive performance. The SCs developed here exhibited a maximum potential voltage of 2.25 V in aqueous H_3_PO_4_ electrolyte, giving a high current density of 24 mA cm^−2^. A high capacitance of 15.8 mF cm^−2^ was observed for a potential window of 1 V. The maximum energy density of 10.22 µW h cm^−2^ at a power density of 11.15 mW cm^−2^ was observed at a current density of 10 mA cm^−2^ for a voltage of 2.25 V. The developed SC shows excellent performance in different static and dynamic bending conditions, which makes them ideal for wearable applications. The fabricated SCs present great cyclic stability in the potential of 2.5 V, without significant deterioration for more than 15 000 cycles of measurement. Finally, we have successfully demonstrated the applicability of fabricated SC in advanced energy storage technologies. In this regard, SCs have been used to: a) operate prosthetic/robotic hand; b) operate fan motors; c) self‐charging power pack (charging through integrated solar cell); and d) wristband for wearable sensors. These results imply that by scaling up the fabrication of SCs and solar‐charging the developed technology has future potential applications in electric vehicles, wearable systems, robotics/prosthetics, and deployment in portable and remote area usage. For some of these applications the mass specific capacitance is also a relevant figure of merit. Our future work will also focus on such figures of merits by investigating SCs with different electrode thicknesses and mass.

## 4 Experimental Section

*Active Electrodes Materials and Fabrication Steps*: GPU composite is used as an active material consisting of graphite (Sigma‐Aldrich) as a conductor phase and PU for agglutinating the graphite. The GPU composite paste in the ratio of 1:1 (wt%) was prepared by mixing the graphite with PU. The graphite powder was ground in an agate mortar and PU were added to prepare the printable paste, shown in Figure 1a. The optimization of the GPU composite was carried out in terms of printability and the conductance of GPU composite electrode. The presence of higher PU concentration in GPU composite (1:2) renders the composite to be nonconducting. However, higher graphite concentration leads to resulting in poor printability of electrode. Hence, in this work a 1:1 ratio of GPU was found to be suitable for this electrode fabrication. The thickness of the commercial GS film is ≈15 µm and for GPU film is ≈90 µm. In this work, for comparing and investigating the electrochemical performance three flexible EDLCs were developed and tested. Comparison of the developed SCs such as GSSC, GPUSC, and GS/GPUSC are shown in Figure 1b. The fabrication steps of GSSC and GPUSC are described with schematic (Figure S6, Supporting Information). The following section describes the fabrication of GS/GPUSC.

GS/GPUSCs was fabricated through a low‐cost printing method schematically described in Figure 1c. i) Flexible polyethylene glycol terephthalate (PET) was used as substrate for fabrication of the SC. In step (ii) a PU resin was drop casted on top of the PET substrate for bonding the conductive multilayer graphene (GS) (Graphene Supermarket US) which functions as a current collector in GS/GPUSC and is shown in step (iii). The mechanically bonded GS on the PET by using PU as an adhesive layer was heat treated at 80 °C for 2 h. After it cools down to room temperature, the prepared GPU composite layer was printed on the top of GS and the sample was heat treated at 80 °C for 2 h (shown in step (iv)). In step (v) a copper strip was attached at the end of GS for collecting the charge stored in SC active electrodes serving as external terminals of the SC. This copper strip was protected from the electrolyte by using an insulating resin. The surface of the electrode was filled with phosphoric acid (H_3_PO_4_) as an electrolyte (presented in step (vi)). H_3_PO_4_ is a promising electrolyte for EDLC based SCs due to the smaller ionic radius of H^+^ (which can diffuse very easily into graphite layers) than that of the ions Na^+^, K^+^, OH^−^, Cl^−^ etc., resulting in free ion concentration in solution than other electrolytes (NaOH or NaCl).^41^ For verifying the influence of electrolyte on the performance of EDLC NaOH was also used as an electrolyte (results are discussed in the Supporting Information). Two identical electrodes are prepared and placed together by using polyester/cellulose blend (Techni Cloth, TX 612) as a separator and medium for keeping the electrolyte, shown in step (vii). A further improvement of the wettability has been achieved by wrapping the above separators around the electrodes of SC (illustrated in step (viii)), which also leads to a further compaction of the SC. In addition to this, the above device architecture also helps reduce the ionic resistance observed in single separator‐based SC as shown by the analysis of this work. The cross‐sectional view of the fabricated GS/GPUSC is shown in step (ix). For flexible applications, the SCs are laminated by using lamination press. No ethical approval was needed for the experiments shown in this manuscript.

*Characterizations*: The morphology of GS and G‐PU films were observed from SEM (SU8240, BRUKER at 15 kV and working distance (WD) of 8 mm) images. The sample was under scanned by stylus profilometer (DektakXT, Bruker). 3D scanning was performed over an area of 500 µm × 500 µm with 100 scans. The surface roughness is calculated by software (Vision 64, Bruker). Surface roughness of the GPU films was investigated by using AFM by using soft‐tapping mode with Dimension Icon AFM from Bruker. For measuring the hydrophobicity, aqueous H_3_PO_4_ electrolyte was drop coated on top of both GS and GPU film surface to study the contact angle of the resulting droplet (θ). The crystal structure of the GPU film was carried out using X‐ray diffractometer (XRD), P'Analytical X'Pert, with Cu *K*_α_ (λ  =  1.514 Å). The crystal and chemical structure of the GS and G‐PU films were investigated by using a Thermo Fisher DXR Raman Microscope (USA) which had a diode‐pumped solid‐state (DPSS) laser with a wavelength of 532 nm. The functional group analysis of the GS and G‐PU composite film were investigated by using an FTIR spectrometer (VERTEX 70, BRUKER).

Electrochemical performances of the as‐prepared SCs were evaluated using CV, electrochemical impedance spectroscopy (EIS) and constant current charge–discharge (GCD) methods in a two‐electrode electrochemical cell system by using Metrohm Autolab (PGSTAT302N, Netherland). CV analysis was carried out at a scan rate of 25–200 mV s^−1^ in a potential range of −1 to 1 V. The EIS measurements were carried out from 10 mHz to 100 kHz at sinusoidal signals of 5 mV. The measured complex impedance data were analyzed by using Nyquist and Bode plot also indicated the series equivalent capacitance variations with frequency. The GCD measurements of the fabricated SCs were tested by using source meter (Agilent, U2722A) controlled with LabVIEW program at different current densities with a potential window 1 and 3.5 V. The maximum operating voltage or saturation voltage (*V*_sat_) was also investigated for analyzing the maximum energy and power density of the device. The value of areal capacitance (*C*_A_), energy (*E*_A_), and power (*P*_A_) densities of the fabricated devices were measured and compared by using GCD measurements at various applied current densities.

The response of GS/GPUSCs were characterized under static and dynamic bending conditions. On one hand, the static characterization of GS/GPUSC performance was carried out by conformably wrapping the GS/GPUSC at top of the surface of 3D‐printed semicylinders (printed in a CubePro Trio) with various radii of 25, 20, and 15 mm. For checking the performance of GS/GPUSC in dynamic bending, the GS/GPUSC connected to two linear stage motors (from Micronix USA). Both motors are synchronized through LabVIEW to move along opposite directions, resulting in a cyclic bending of the GS/GPUSC at bending radius of 24 mm and at a speed of 0.5 mm s^−1^ (Movie S1 attached the demonstration of cyclic bending in the Supporting Information).

## Conflict of Interest

The authors declare no conflict of interest.

## Supporting information

Supplementary

Supplementary

Supplementary

Supplementary

Supplementary

Supplementary
