# Supplementary material for: Graphene–Graphite Polyurethane Composite Based High‐Energy Density Flexible Supercapacitors
Source: Adv Sci (Weinh). 2019 Feb 13;6(7):1802251. doi: 10.1002/advs.201802251 (PMC6446598; doi:10.1002/advs.201802251)
Supplement: Supplementary file 1 — Supplementary [file ADVS-6-1802251-s002.pdf]

Copyright WILEY-VCH Verlag GmbH & Co. KGaA, 69469 Weinheim, Germany, 2019.

# Supporting Information

for *Adv. Sci.,* DOI: 10.1002/advs.201802251

# Graphene–Graphite Polyurethane Composite Based High-Energy Density Flexible Supercapacitors

*Libu Manjakkal, William Taube Navaraj, Carlos García Núñez, and Ravinder Dahiya**

WILEY-VCH

Copyright WILEY-VCH Verlag GmbH & Co. KGaA, 69469 Weinheim, Germany, 2019.

# Supporting Information

## Graphene-Graphite Polyurethane Composites based High-Energy Density Flexible Supercapacitors

*L. Manjakkal, W. T. Navaraj, C. G. Núñez and R. Dahiya**

**Table S1.** Comparison of the areal capacitance between the GSC and several reported SC based on graphene

| **Material** | **Electrolyte** | **Operating potential (V)** | **Areal Capacitance ($C_A$) mF.cm$^{-2}$** | **Ref** |
|---|---|---|---|---|
| Flexible 3D-graphene/ graphite-paper (full cell) | PVA-$H_2SO_4$ | 1 | 9 at 0.05 mA | [1] |
| Electrochemically activated reduced graphene oxide (rGO) film | PVA/$H_3PO_4$ | 0.8 | 11.15 at 1 mA.cm$^{-2}$<br>15.38 at 0.1 mA.cm$^{-2}$ | [2] |
| Onion-like carbon | 1M $Et_4NBF_4$/ Anhydrous propylene carbonate | 3 | 1.7 at 1 V s$^{-1}$ | [3] |
| Nitrogen-doped rGO (flexible) | PVA/$H_3PO_4$ | 0.8 | 3.4 at 20 µA cm$^{-2}$ | [4] |
| Hydrated graphite oxide | 1.0 M $Na_2SO_4$ and 1.0 M $TEABF_4$ in acetonitrile | 1 | 0.51 | [5] |
| Ink-jet printed carbon | 1 M $Et_4NBF_4$ propylene carbonate | 2.5 | 2.1 at 0.001 V s$^{-1}$ | [6] |
| Interdigital graphene | $H_2SO_4$–PVA | 1 | 0.116 at 0.01 V s$^{-1}$ | [7] |
| rGO | PVA/$H_2SO_4$ | 2 electrodes | 0.95 at 0.43 mA.cm$^{-2}$ | [8] |
| rGO/CNT | 3 M KCL | 1 | 6.1 at 0.01 V s$^{-1}$ | [9] |
| Laser-induced Graphene | $H_2SO_4$/PVA | 1 | > 9 at 0.02 mA.cm$^{-2}$ | [10] |
| Graphene sheet-Graphite -PU composite based flexible electrode | $H_3PO_4$ | 2.25 | 14.79 at 10 mA.cm$^{-2}$ | This work |

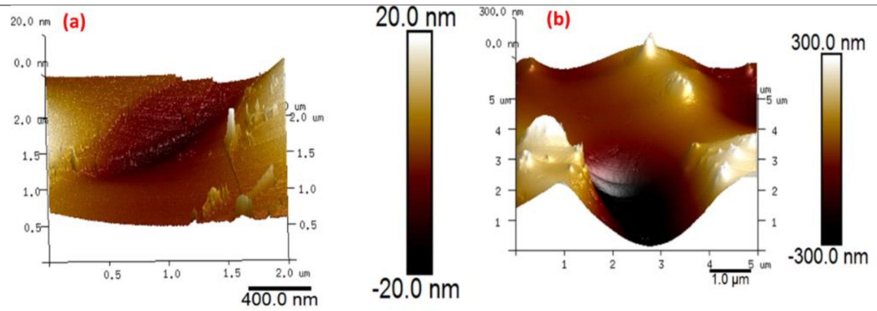


Figure S1: (a) and (b) 3D AFM images of GS and GPU films.

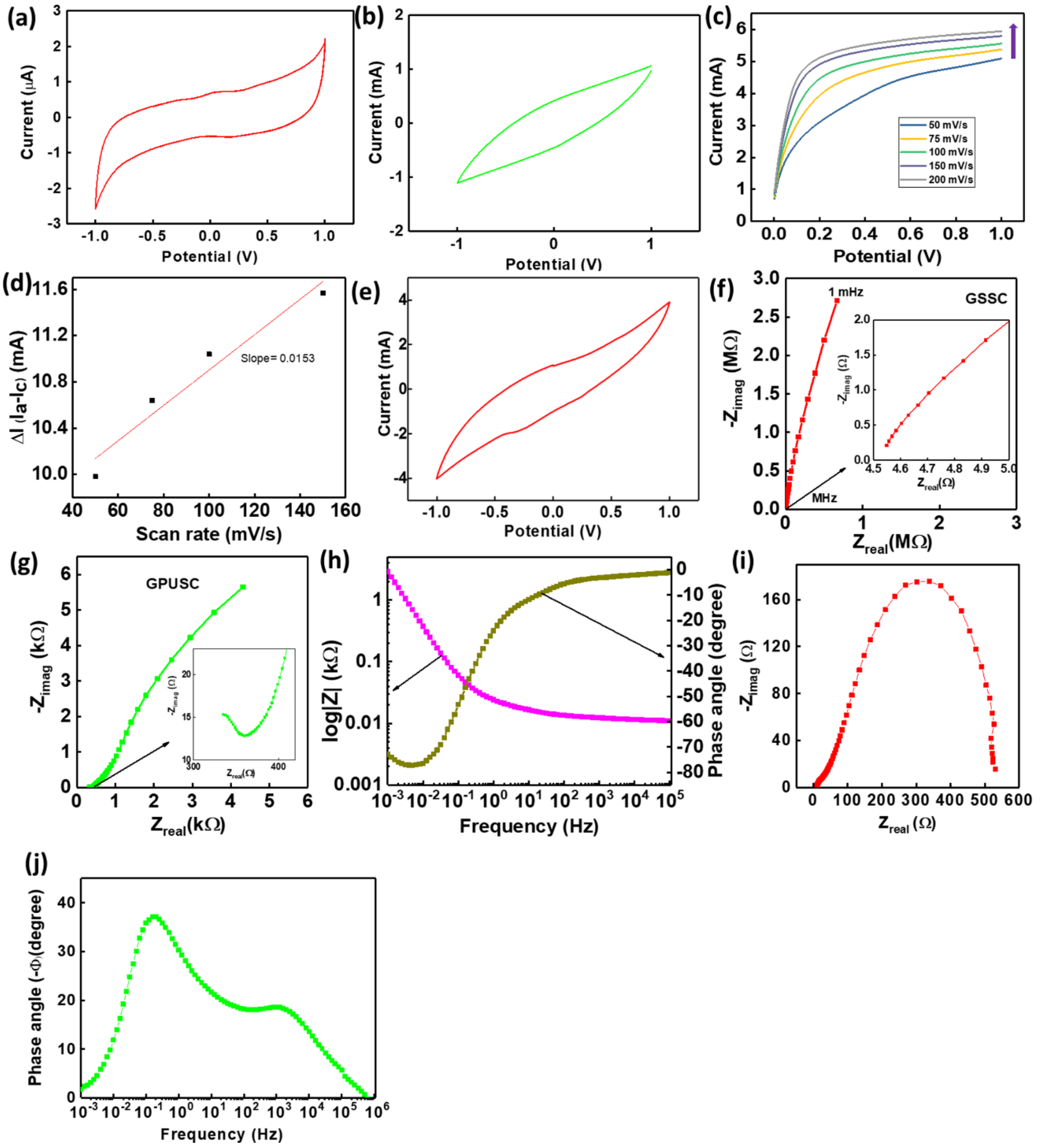


Figure S2: (a) and (b) CV profile of GSSC and (b) GPUSC at a scan rate 100 mV.s$^{-1}$. (c) I-V curves of GS/GPUSC measured at various scan rates (d) current density differences vs the scan rate for measuring double layer capacitance for ESCA measurement (e) CV profile of single layer separator GS/GPUSC at a scan rate of 100 mV. s$^{-1}$. (f) and (g) Nyquist's plot of GSSC and GPUSC. (h) Bode impedance and phase angle plot for GS/GPUSC (i) Nyquist and (j) Bode plot for GS/GPUSC using single separator.

As compared to GS/GPUSC with separator wrapped around the active electrodes (GS/GPUSC -1), the single separator GS/GPUSC shows a significant change in the value resistance and reactance. Instead of straight line in Nyquist plot in low frequency range (Figure 4) of wrapped separator in GS/GPUSC, the single separator-based device has a semicircle arc in the low frequency range, Figure S2i. It potentially due to the ion adsorption rather than diffusion in low frequency range. The configuration of GS/GPUSC have strong influence on the value of capacitance.  It was observed that, in low frequency ionic resistance have strong influence

and hence phase angle reach to zero. Moreover, the maximum value of phase angle observed for single layered separator GS/GPUSC is -37° and is very low as compared to wrapped separator GS/GPUSC (-77°). This shows that electrochemical and capacitive performance of the device is poor.

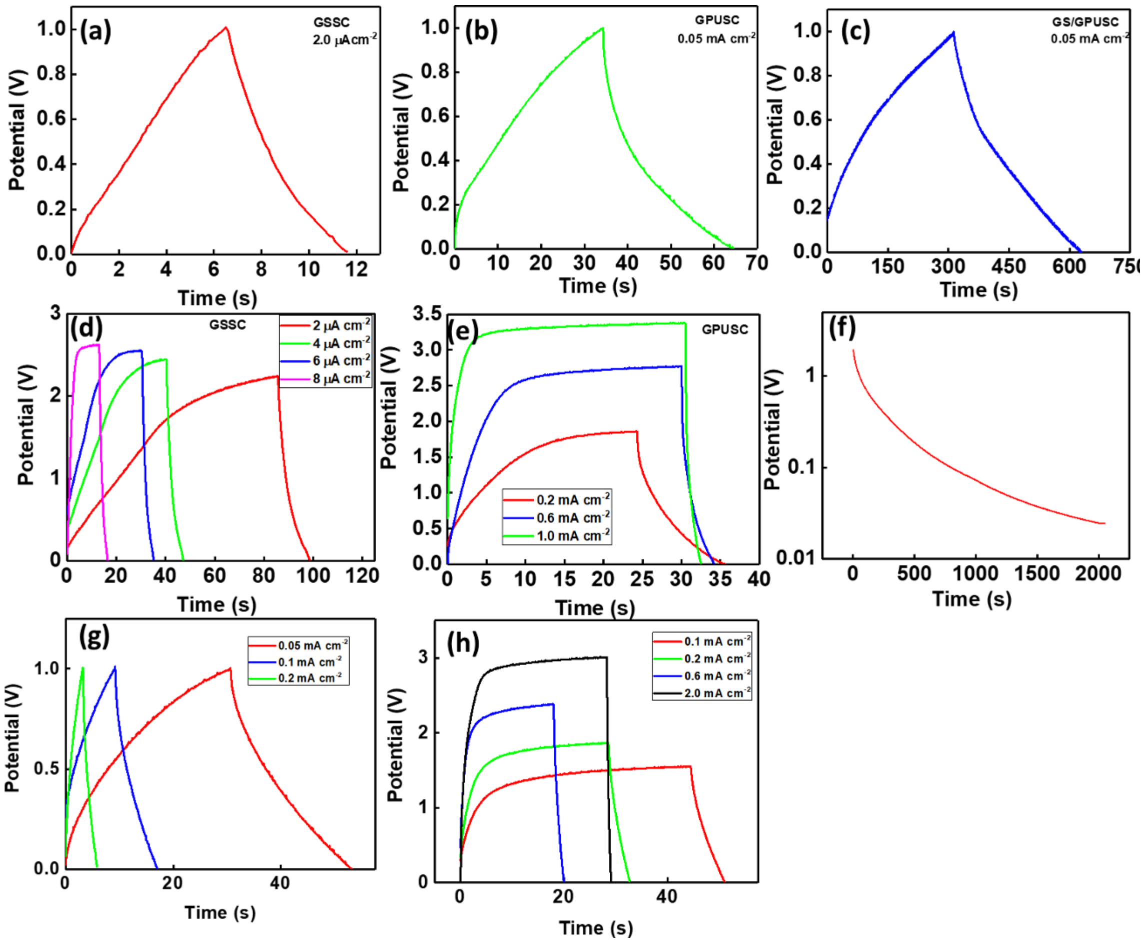


Figure S3: GCD curves for (a) GSSC, (b) GPUSC and (c) GS/GPUSC at the potential window of 0-1V. (d) and (e) GCD curves for saturation potential of GSSC and GPUSC respectively. (f) Self-charging of the GS/GPUSC (g) GCD curve at fixed potential window of GS/GPUSC based on NaOH electrolyte (h) GCD curve for saturation potential of GS/GPUSC based on NaOH electrolyte.

***Comparison of Charging-Discharging analysis of SCs***

In the case of GSSC, the charging-discharging occurred at very low current densities (in the range of 2-8 μA $cm^{-2}$), as shown in Figure S3d for $V_{sat}$. Figure S3d and S3e shows the GCD measurements carried out in GSSC and GPUSC to obtain the $V_{sat}$. It is worth noticing that even though the GSSC and GPUSC can reach $V_{sat}$ between 2 - 3V, using high current densities, both SCs discharge at a faster rate than GS/GPUSC. As a result, the applicability of GSSC and GPUSC will be limited. The poor operation at high current density and the quick discharging of these devices can be overcome by using the double-layered structure as in the case of GS/GPUSCs.

***Role of Electrolyte on SCs Performance***

From EIS and CV analysis, we have observed that the SC cell configuration with separator also plays a crucial role towards the enhancement of the electrochemical performance of the GS/GPUSCs. In addition, to evaluate the influence of electrolyte on the

performance of the SCs, we have carried out GCD measurements for GS/GPUSC with NaOH electrolyte and compared with results obtained by using $H_3PO_4$ electrolyte (Figure S3g and S5h and comparison under different current densities in Table S2). We noted that, as compared to $H_3PO_4$, the NaOH shows faster discharging times, which could be due to low ionic concentration for EDL formation. The $H^+$ ions in $H_3PO_4$ can diffuse more efficient into GPU matrix than $Na^+$ ions from NaOH electrolyte.

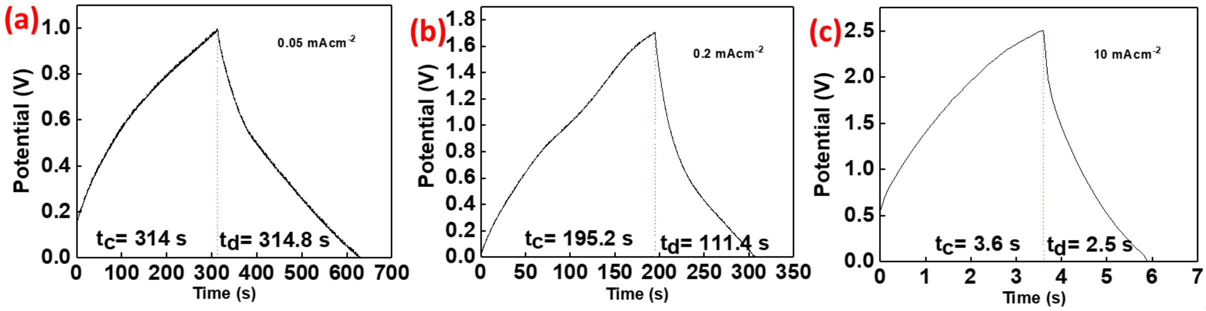


Figure S4: Charging and discharging time for different current densities (a) at 0.05 $mA.cm^{-2}$ for fixed potential window of 1V. (b) at 0.2 $mA.cm^{-2}$ for $V_{sat}$ and (c) for high current density of 10 $mA.cm^{-2}$.

***Operational efficiency of SCs***

The operational efficiency or performance of the SCs, is determined thorough the areal capacitance ($C_A$), energy ($E_A$) and power ($P_A$) densities, the equations (1) to (3).

The specific capacitance ($C_A$) of the SC at different current densities were obtained from the following equation[11]

$$C_A = \frac{I \times \Delta t}{\Delta V \times A} \quad \ldots\ldots\ldots\ldots\ldots\ldots\ldots\ldots\ldots\ldots \ (1)$$

Where I is the discharge current Δt is the discharge time of the electrochemical capacitor, ΔV is the potential window (here we consider 0 to 1V and 0 to $V_{sat}$) and A is the area of active material.

The energy and power density of the fabricated GSC were calculated by using

$$E_A = \frac{1}{2} C_A \Delta V^2 \quad \ldots\ldots\ldots\ldots\ldots\ldots\ldots\ldots\ldots \ (2)$$

$$P_A = \frac{E_A}{\Delta t} \quad \ldots\ldots\ldots\ldots\ldots\ldots\ldots\ldots\ldots\ldots\ldots \ (3)$$

The specific capacitance ($C_A$) of the SC can be calculated with IR voltage drop by using the equation

$$C_A = \frac{I \times \Delta t}{\Delta V - IR_{drop} \times A} \quad \ldots\ldots\ldots\ldots\ldots\ldots\ldots\ldots \ (4)$$

Where $IR_{drop}$ is the IR drop voltage at high current densities.
The energy and power density of the fabricated GSC were calculated by using

$$E_A = \frac{1}{2} C_A (\Delta V - IR_{drop})^2 \quad \ldots\ldots\ldots\ldots\ldots\ldots\ldots \ (5)$$

$$P_A = \frac{E_A}{\Delta t} \quad \ldots\ldots\ldots\ldots\ldots\ldots\ldots\ldots\ldots\ldots\ldots \ (6)$$

Table S2: Comparison of performance of GS/GPUSC in NaOH solution in potential window

| **Current density** | **Areal Capacitance** | **Energy density** | **Power density** |
|---|---|---|---|

| **(mA.cm$^{-2}$)** | **mFcm$^{-2}$** | **µW.h.cm$^{-2}$** | **mW.cm$^{-2}$** |
|---|---|---|---|
| 0.05 | 1.13 | 0.15 | 0.023 |
| 0.10 | 0.78 | 0.10 | 0.057 |
| 0.20 | 0.52 | 0.07 | 0.097 |

Table S3: Comparison of energy and power density carbon based supercapacitor

| **Material** | **Electrolyte** | **Energy density** | **Power density** | **Ref** |
|---|---|---|---|---|
| Flexible 3D-graphene/ graphite-paper | $H_2SO_4$ | 1.24 µW.h.cm$^{-2}$ | 24.5 µW.cm$^{-2}$ | 1 |
| Graphene thin film | PVA-$H_3PO_4$ | 0.235 µW.h.cm$^{-2}$ | 0.106 mW.cm$^{-2}$ | 12 |
| Ultrathin CVD graphene films | $H_2SO_4$-PVA | 0.27 nW h cm$^{-2}$ | 36.48 µW.cm$^{-2}$ | 13 |
| Polypyrrole/graphene oxide (PPy/GO) | KCl | 12.9 µW.h.cm$^{-2}$ | 954.3 µW.cm$^{-2}$ | 14 |
| Polyaniline (PANI)/graphite oxide (GO) nanocomposite films | $H_2SO_4$ | 2.52 µW.h.cm$^{-2}$ | 0.01 mW.cm$^{-2}$ | 15 |
| Ultrathin Planar Graphene | $H_3PO_4$ | 2.8 nW h cm$^{-2}$ | 2 µW.cm$^{-2}$ | 16 |
| Flexible fiber supercapacitor | $Na_2SO_4$ | $2.70\times10^{-6}$ to $1.76\times10^{-6}$ W h cm$^{-2}$ | 0.04 - 9.07 mW.cm$^{-2}$ | 17 |
| Carbon Nanotube/$MnO_2$/Polymer Fiber | PVA-LiCl | 2.6 µW.h.cm$^{-2}$ | 66.9 µW.cm$^{-2}$ | 18 |
| $MnO_2$ Coated Carbon Nanotube | KOH | 1.14 µW.h.cm$^{-2}$ | 1.50 mW.cm$^{-2}$ | 19 |
| Three-dimensional (3D) graphene oxide/polypyrrole (GO/PPy) composite | KCl | 16.8 µW.h.cm$^{-2}$ | 0.08 mW.cm$^{-2}$ | 20 |
| electrochemically expanded graphite foil | $H_2SO_4$ | 0.163 mW h cm$^{-3}$ and 0.124 mW h cm$^{-3}$ | 19 mW.cm$^{-3}$ and 447 mW.cm$^{-3}$ | 21 |
| N-doped reduced graphene oxide (rGO) | PVA - $H_3PO_4$ | 3.0 x $10^{-4}$ W h cm$^{-3}$ | 0.2 W.cm$^{-3}$ | 4 |
| hydrated graphite oxide films | $Na_2SO_4$ | 4.3 × $10^{-4}$ W h cm$^{-3}$ | 1.7 W.cm$^{-3}$ | 22 |
| Laser Scribed Graphene From GO | 1-ethyl-3-methylimidazolium tetrafluoroborate (EMIMBF4) | 1.36 mW h cm$^{-3}$ | 20W.cm$^{-3}$ | 23 |
| Reduced Graphene Oxide and Carbon Nanotube | KCl | ~0.68 mW h cm$^{-3}$ | 77 W.cm$^{-3}$ | 9 |
| Graphene sheet-Graphite -PU composite based flexible electrode | $H_3PO_4$ | 10.22 µW.h.cm$^{-2}$ 0.567 mW h cm$^{-3}$ | 11.15 mW.cm$^{-2}$ 618 mW.cm$^{-3}$ | This work |

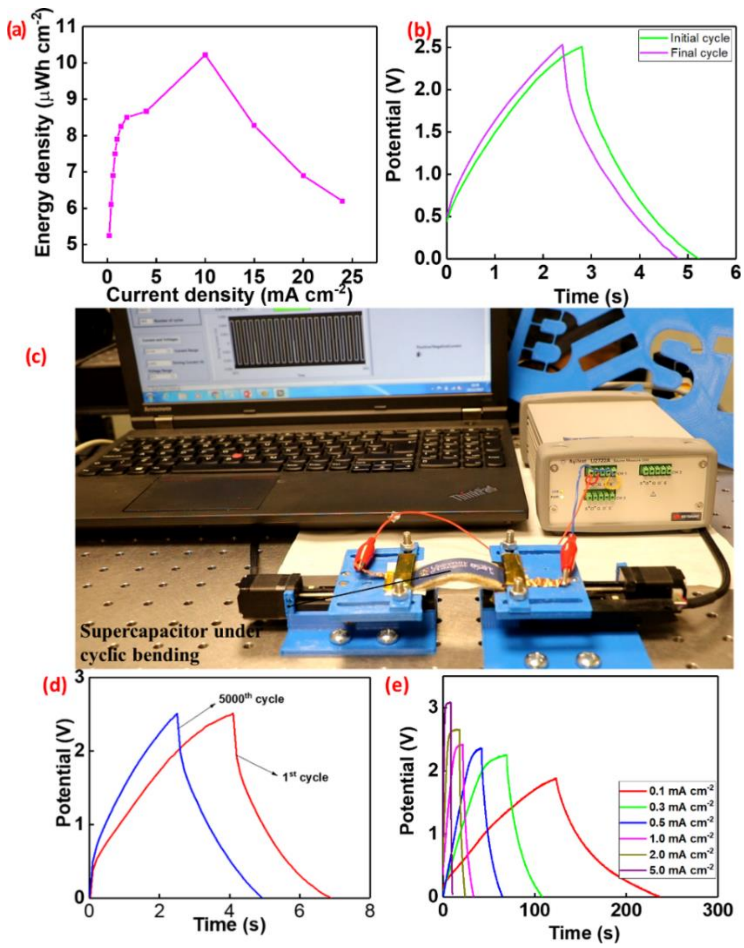


Figure S5: (a) The performances of energy density of GS/ at high and low current densities for $V_{sat.}$ (b) GCD curve of the initial and $10000^{th}$ cycles (c) Image of GS/GPUSC under cyclic bending (d) GCD curve for initial and final cycle of 5000 cycles after measuring 10000 cycle. (e) Charge and discharge curves for wristband G-GGSC for $V_{sat.}$

Table S4: Comparison of charging and discharging of the device under 15000 cycles of operation

| **Time** | **1$^{st}$ cycle** | **15000$^{th}$ cycle** |
|---|---|---|
| Charging time | 2.8 s | 2.5 s |
| Discharging time | 2.5 s | 2.5 s |

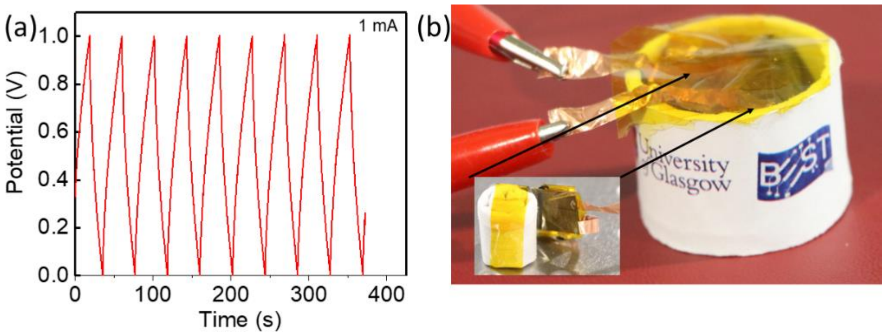


Figure S6: (a) GCD curve for the SC on the (b) inner surface of a 3D printed cylinder

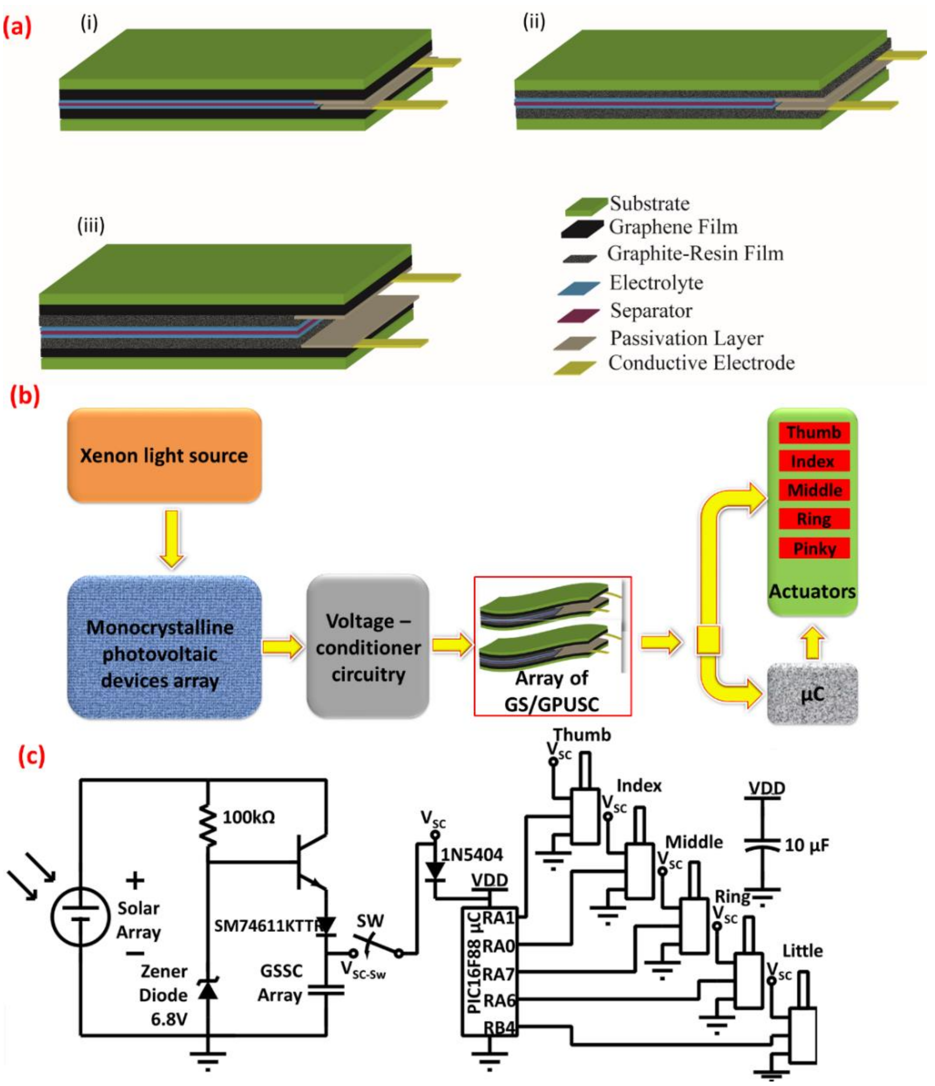


Figure S7: (a) Comparison of cross-sectional schematic representation of (i) GSSC, (ii) GPUSC with (iii) GS/GPUSC. (b) Block diagram for solar-powered 3D-printed prosthetic hand application. (c) Voltage-regulator circuit for the prosthetic hand application of SC using solar power.

***Applications***

For the wearable applications of the fabricated GS/GPUSC were converted as a wristband. The fabricated electrodes are well sealed and packed by using lamination. The PVC sheet was used for the encapsulation of the device. Wristband based SC connected to 5 LEDs. The fabricated wristband has future potential application for energy supply to the flexible physical and chemical sensors and also for wearable electronics in e-skin applications. We have demonstrated the applicability of the developed SC as an energy storage device in a solar-charged power pack system. Fabricated GS/GPUSCs were integrated to a poly-crystalline Si (poly-Si) photovoltaic (PV) cell. The fabricated SCs were packed in 3D printed boxes. Firstly, we have connected the SCs to the PV cell for charging, and then through an electrical current switch, we have connected the SCs to 84 LEDs (modelled as a letter of BEST and UoG) as

illustrated. SCs are charged by using a poly-Si PV cell under 1 sun illumination, resulting in a charging current of 10 mA, which was sufficient to reach a potential window of 5 V (2.5 V each SC cell). As presented in Movie 2 (Supporting Information), the solar-charged power pack system demonstrated capability to power 84 for more than 1 min. In addition, the long lifetime (15000 charging/discharging cycles) of the proposed SCs and its performance in solar charging demonstrate its potential application for deployment in portable and remote area usage.

To further demonstrate the application of SC for a high-power density application, the fabricated flexible GS/GPUSC have been connected to a motor which has an operating voltage range of 1.5 to 4.5V DC. The motor was connected to a fan with plastic blades. The motor has a speed of 2300 RPM at 4.5VDC in 70mA without any load. Here, we connected three GS/GPUSC in parallel for operating a single motor. The SCs were charged by using 120 mA current at 2.5V. After charging, the SCs were discharged by switching the motor connected to the fan. The performance of the SCs for single motor operation is shown in Movie 3 in Supporting Information. For operating 3 such motors and propeller fan we connected 9 such GS/GPUSC in parallel as shown in Movie 4 in Supporting Information. These motors are normally used in electric toy cars. The demonstration of SCs implies that by scaling up the fabrication of SCs and solar-charging the developed technology has future potential applications in electric vehicles. The developed SCs have also been used as an energy storage device in a solar-powered prosthetic/robotic hand. The SCs serves as a buffer to offer higher current discharge during the operation of various motors of the prosthetic hand, which is not possible with solar cells alone. Together with solar cells, the SCs offer an attractive solution for future energy autonomous robotics and prosthetics. Energy autonomy is currently a major challenge in autonomous robotics and prosthetics. The block diagram and the electrical schematic of the implemented system is shown in Figure S6 in Supporting Information.

The prosthetic hand, attached to the shoulder of dummy, is based on an in-house design and was developed by 3D printing ABS. In the current implementation, all the finger actuators are powered by the PV cells and SCs while the elbow and shoulder motors are not used. A total of 5 micro linear servos (PQ12-R) are used to control the open and close movements and positions of each fingers. The power consumption of the motor varies from 50 mA under no load, 250 mA at 50 N load to maximum current of 550 mA when stalled. Monocrystalline silicon PV cells of 2.75 cm x 12 cm x 100 µm with nominal efficiency of 15.4% has been assembled on the faces of a semi-flexible sheet bent into an octagonal hollow cylinder. The

radius of curvature of the octagonal-bent cylinder is 4 cm. A total of 16 cells were assembled in series. When the cell array is in flat condition, with AM1.5 equivalent incident spectral illumination from a single xenon lamp, the panel gives a maximum power point output voltage ($V_{mp}$) of ~8 V. However, with bent condition of this solar cell assembly, the voltage level decreases to 7.6 V in the condition shown in the Movie 5 in Supporting Information with a double illumination from opposite angles. This decrease is due to slanted and scattered incidence of the light source resulting in lower illumination intensity per unit area. The GS/GPUSCs were connected in series to achieve storage capability of 6 V. This series array of SCs is wrapped around the forearm of the prosthesis. The output of the solar cell array has been fed to the GS/GPUSC array through a voltage-regulator circuit as shown in Figure S7 in Supporting Information where the regulation voltage is determined by the Zener diode (TZX6V8C-TR), the drop in the base emitter (~0.65V) and the diode (SM74611KTTR). As shown in the Movie 5 (in Supporting Information), after the SC gets charged, a switch (SW) is toggled to turn on the prosthesis. This results in the SC powering all the micro servo motors. A diode 1N5404 is used to lower the VSC supply voltage to suit the 1688 microcontroller's preferred operating voltage of 4.0 V to 5.5 V. The microcontroller is programmed to output pulse width modulated (PWM) signal to control the position of the various servos on various pins according to requirement.  In the Movie 5 (in Supporting Information), the index and thumb fingers are actuated through PWM to perform grab and release action. The presented demonstration is the first time SCs and solar cells are used to power a prosthetic hand GS/GPUSC.

**References**


1. A. Ramadoss, K.-Y. Yoon, M.-J. Kwak, S.-I. Kim, S.-T. Ryu, J.-H. Jang, J. Power Sources, 337, (2017) 159-165.
2. M. Wu, Y. Li, B. Yao, J. Chen, C. Li, G. Shi, J. Mater. Chem.  A,  4 (2016) 16213-16218.
3. D. Pech, M. Brunet, H. Durou, P. Huang, V. Mochalin, Y. Gogotsi, P.-L. Taberna, P. Simon, Nat Nano, 5 (2010) 651-654.
4. S. Liu, J. Xie, H. Li, Y. Wang, H. Y. Yang, T. Zhu, S. Zhang, G. Cao and X. Zhao, J. Mater. Chem. A, 2, (2014)18125-18131.
5. W. Gao, N. Singh, L. Song, Z. Liu, A. L. M. Reddy, L. Ci, R. Vajtai, Q. Zhang, B. Wei, P. M. Ajayan, Nat Nano, , 6 (2011) 496-500.
6. D. Pech, M. Brunet, P.-L. Taberna, P. Simon, N. Fabre, F. Mesnilgrente, V. Conédéra, H. Durou, J. Power Sources, 195, (2010) 1266-1269.
7. J. Wu, Y. Li, Q. Tang, G. Yue, J. Lin, M. Huang and L. Meng, Sci. Rep. 4. (2014)
8. Z.-K. Wu, Z. Lin, L. Li, B. Song, K.-s. Moon, S.-L. Bai, C.-P. Wong, Nano Energy, 10, (2014) 222-228.
9. M. Beidaghi, C. Wang, Adv. Funct. Mater. 22, (2012) 4501-4510.
10. Z. Peng, J. Lin, R. Ye, E. L. G. Samuel and J. M. Tour, ACS Appl. Mater. Inter. 7, (2015) 3414-3419.
11. K. Wang, X. Dong, C. Zhao, X. Qian, Y. Xu, Electrochimica Acta, 152 (2015) 433-442.

12. Q. Chen, X. Li, X. Zang, Y. Cao, Y. He, P. Li, K. Wang, J. Wei, D. Wu, H. Zhu, RSC Advances, 4, (2014) 36253-36256.
13. P. Xu, J. Kang, J.-B. Choi, J. Suhr, J. Yu, F. Li, J.-H. Byun, B.-S. Kim, T.-W. Chou, ACS Nano, 8, (2014) 9437-9445.
14. H. Zhou, G. Han, Y. Xiao, Y. Chang and H.-J. Zhai, J. Power Sources, 263 (2014) 259-267.
15. H. Wei, J. Zhu, S. Wu, S. Wei, Z. Guo, Polymer, 54 (2013)1820-1831.
16. J. J. Yoo, K. Balakrishnan, J. Huang, V. Meunier, B. G. Sumpter, A. Srivastava, M. Conway, A. L. Mohana Reddy, J. Yu, R. Vajtai, P. M. Ajayan, Nano Letters, 11, (2011) 1423-1427.
17. Y. Fu, X. Cai, H. Wu, Z. Lv, S. Hou, M. Peng, X. Yu, D. Zou, Adv. Mater., 24, (2012) 5713-5718.
18. C. Choi, S. H. Kim, H. J. Sim, J. A. Lee, A. Y. Choi, Y. T. Kim, X. Lepró, G. M. Spinks, R. H. Baughman, S. J. Kim, Sci. Rep., 5, (2015) 9387.
19. P. Xu, B. Wei, Z. Cao, J. Zheng, K. Gong, F. Li, J. Yu, Q. Li, W. Lu, J.-H. Byun, B.-S. Kim, Y. Yan, T.-W. Chou, ACS Nano, 9 (2015) 6088-6096.
20. J. Cao, Y. Wang, J. Chen, X. Li, F. C. Walsh, J.-H. Ouyang, D. Jia, Y. Zhou, J. Mater. Chem. A, 3 (2015) 14445-14457.
21. H.-Y. Li, Y. Yu, L. Liu, L. Liu, Y. Wu, Electrochimica Acta, 228 (2017) 553-561.
22. W. Gao, N. Singh, L. Song, Z. Liu, A. L. M. Reddy, L. Ci, R. Vajtai, Q. Zhang, B. Wei, P. M. Ajayan, Nature Nanotechnol. 6 (2011) 496.
23. M. F. El-Kady, V. Strong, S. Dubin, R. B. Kaner, Science, 335, (2012) 1326-1330.

**Supplementary Movie Notes**

**Supplementary Movie-1**
Stable and reliable operation of flexible supercapacitors is crucial for several applications. In this regard, we have demonstrated the applicability of fabricated GS/GPUSC in cyclic bending with a radius of 24 mm as an example.

**Supplementary Movie-2**
The proposed supercapacitors and its performance in solar charging demonstrate its potential application for deployment in portable and remote area usage. We demonstrate this through integrating supercapacitor with solar cell and powered to 84 LEDs, which is modelled as a letter of BEST and UoG.

**Supplementary Movie-3**
The operating voltage of ~2.5 V allows the SCs power general-purpose electronics and hence makes them suitable for a wide range of applications. As a demonstrations 3 supercapacitors connected to a single motor (normally used in electric toy cars) has a speed of 2300 RPM at 4.5VDC in 70mA without any load. The SCs were charged by using 120 mA current at 2.5V.

**Supplementary Movie-4**
The performance of the SCs (9 SCs connected) for three motors operation is shown in this movie.

**Supplementary Movie-5**
Energy autonomy is currently a major challenge in autonomous robotics and prosthetics. Together with solar cells, the SCs offer an attractive solution for future energy autonomous robotics and prosthetics. In this movie, the index and thumb fingers are actuated through PWM to perform grab and release action.
